# A Review of the Effectiveness of Foreign Language Enjoyment and Foreign Language Classroom Anxiety on Learners' Engagement and Attainment

**DOI:** 10.3389/fpsyg.2021.749284

**Published:** 2021-09-06

**Authors:** Jingping Shen

**Affiliations:** School of Foreign Languages, Xinxiang University, Xinxiang, China

**Keywords:** positive psychology, foreign language enjoyment, foreign language classroom anxiety, second language acquisition, success, engagement, attainment

## Abstract

Cognitive factors are not the fundamental determinants of success in language learning. Foreign language attainment depends on both cognitive and affective factors, highlighting the deeper impacts of the former. Some scholars started to investigate affective issues, particularly negative emotions in language learning studies; nevertheless, reducing negative emotions such as anxiety should be accompanied by the development of positive emotions (e.g., well-being, autonomy, and enjoyment). Since then, a great number of researchers have examined the impact of anxiety and enjoyment in foreign language literature, particularly after the introduction of reliable and valid foreign language classroom anxiety (FLCA) and foreign language enjoyment (FLE) scales. So, the present study aims to review contemporary scholarly articles and books in this regard. Findings suggest that there has been a major interest in the evaluation of FLCA and FLE across a variety of dimensions including personality traits, interpersonal characteristics, and classroom conditions. The central issues are summarized into three categories of the relationship between FLCA and FLE, the robustness of respective scales, and the impact of individual and interpersonal factors. Hence, this research attempts to highlight probable gaps and areas for further examinations to help enrich the literature and improve the theoretical knowledge.

## Introduction

The present review study aims to explore foreign language classroom anxiety (FLCA) and foreign language enjoyment (FLE) as fundamental emotional factors in the process of (foreign) language learning. The introduction of the concept of “affective filter” by Krashen (1985) led language learning scholars to consider negative affective factors in the classroom as they can demotivate language learners. Consequently, MacIntyre and Gardner (1994) argued that anxiety might result in students' poor performance in the classroom and imperfect language learning. Horwitz et al. (1986) development of the Foreign Language Classroom Anxiety Scale (FLCAS) prepared the ground for more systematic investigations of anxiety in FL settings, and they further asserted that assessment anxiety, fear of communication, and negative appraisal apprehension were the main causes of FLCA. Since then, there has been a great body of research on FLCA from various perspectives e.g., language cognition (MacIntyre and Gardner, 1994), FL production (Matsuda and Gobel, 2004), individual characteristics (Liu, 2006), etc.

On the other hand, Seligman and Csikszentmihalyi (2000) claimed that the attainment of success should be regarded as a prominent objective in people's lives. They further argued that positive psychology (PP) highlights the effects of happiness, well-being, and creativity on individuals (Oxford, 2016). Therefore, some studies were conducted on the concept of PP and its implications on people's occupation, education, and social lives (Wang et al., 2021). MacIntyre and Gregersen (2012) as well as Lake (2013) were among the pioneering scholars who emphasized the implementation of PP features in language teaching and learning. As a response to the multitude of studies on negative emotions such as anxiety, Dewaele and MacIntyre (2014) proposed that it is time to consider positive emotions in the language learning profession. They also asserted that employing PP in second/foreign language classrooms can result in the development of motivation, enjoyment, as well as persistence, and ultimately long-standing acquisition of FL.

This review study is intended to analyze the existing literature on FLCA and FLE to explore the research methodologies, theoretical foundations, and empirical findings accordingly.

## Theoretical Background

### FLCA as an Affective Factor

Language learning practitioners traditionally believed that it was a process of cognitive engagement in perceiving and producing the target language to convey the meaning. However, since the 1980s, many scholars have highlighted the significant role of affective factors along with cognitive capabilities that can lead to successful FL attainment. Scholars have focused on the impact of a number of negative factors (emotions) in language learning experiences and outcomes, including attitudes toward teachers, boredom, and anxiety (Elahi Shirvan and Talebzadeh, 2020; Derakhshan et al., 2021a).

According to Horwitz et al. (1986), language learning classroom anxiety stems from complicated feelings, understandings, and behaviors pertaining to the process of language learning. Findings of a review study by Young (1999) indicated six sources of language learning anxiety including classroom processes, teacher-learner interactions, individual and interpersonal issues, language testing, learners' attitudes toward language learning, and teachers' beliefs about learning process. Moreover, FLCA was perceived as “the classroom related apprehension and stress,” which commonly happens while performing FL tasks such as speaking activities (MacIntyre and Gardner, 1994, p. 284).

Dewaele et al. (2018) concluded that lower scores in FLCA are associated with higher levels of attitudes toward the target language and successful FL learning; consequently, it is necessary to observe and identify primary causes of language learners' anxiety in the classroom and then reduce FLCA to help them flourish in their language learning process.

### FLE Represents Positive Psychology

With the advent of positive psychology (PP) whose primary objective was to help people flourish and lead them toward success in their lives, Seligman and Csikszentmihalyi (2000) highlighted a crucial gap in the literature (Gabryś-Barker and Gałajda, 2016). They claimed that the existing studies in second/foreign language learning have not examined the influence of positive individual and interpersonal factors (e.g., creativity, perseverance, hope, autonomy, enjoyment, etc.) accordingly (Csikszentmihalyi and Larson, 2014, pp. 279).

Fredrickson (2001) proposed that FL learners can broaden their understanding and proficiency in the classroom and build personal and social resources to achieve linguistic goals (MacIntyre and Gregersen, 2012). Dewaele and MacIntyre (2016) concluded that FLE occurs within three main dimensions of positive setting (feeling of fulfillment from the classroom), positive private (internal or personal feeling of satisfaction), and positive atmosphere (pleasant relationship with teachers and/or peers).

Findings of a seminal study by Pekrun et al. (2007) revealed that learners may experience positive and constant engagement in classroom activities if they perceive they can perform appropriately in classroom tasks, which is regarded as an instance of FLE. Moreover, the PERMA model, developed by Seligman (2018) acknowledged that well-being depends on positive emotions, engagement, relationships, meaning, and achievement. A relatively recent study by Li (2021) systematically reviewed the concepts of psychological well-being, mindfulness, and immunity of teachers in the domain of EFL/ESL educational contexts. Therefore, language learning scholars are recommended to investigate FLE in the academic setting so that learners can develop and improve necessary requirements that lead to success.

## Empirical Studies

The majority of empirical studies on FLCA and FLE can be summarized into the following headings:

### The Relationship Between FLCA and FLE

MacIntyre and Gregersen (2012) argued that positive and negative emotions belong to the same dimension of language learning, but these two concepts are not necessarily opposite. Furthermore, findings of significant research by Dewaele and MacIntyre (2014) reported FLCA and FLE are adversely correlated. However, a single learner can report high levels of FLCA along with high levels of FLE. Hence, it is imperative to investigate these two concepts using different scales within the same conceptual category.

Dewaele et al. (2018) conducted a study on 189 high school students. The learners were asked to complete a 10-item questionnaire (extracted from Dewaele and MacIntyre, 2014 FLE Scale) along with an 8-item questionnaire (extracted from Horwitz et al., 's, 1986 FLCA scale). The authors concluded that there is a minor, but significant negative relationship between FLE and FLCA.

### The Robustness of the FLCA and FLE Scales

Horwitz et al. (1986) proposed that foreign language classroom anxiety is associated with language classrooms pertinent to feelings, perceptions, and behaviors. Consequently, they developed the FLCAS to measure learners' anxiety in foreign language classrooms based on three main domains of test anxiety, communication apprehension, and fear of negative appraisal. The original scale consists of 33 items including 24 positively worded and 9 negatively worded statements that are scored based on a 5-point Likert scale (1 = I strongly disagree and 5 = I strongly agree). The total score of the questionnaire would range between 33 and 165. Moreover, the reliability of the scale was assessed and confirmed using internal consistency (Cronbach's alpha coefficient of 0.93). Furthermore, Jin et al. (2020) used the Chinese version of FLCAS to observe the impact of interventions on the reduction of anxiety among 42 language learners. They reported a significant decrease in the classroom anxiety scores after the intervention.

Given that, Dewaele and MacIntyre (2014) attempted to investigate the relationship between FLE and FLCA among 1,746 language learners from different countries, they developed a standard scale to measure FLE. The proposed scale included 21 items pertaining to interest, creativity, fun, and self-importance a well as teachers and peers' impact in the FL classroom. They further modified this scale in 2016; the newly developed questionnaire contained 14 items and the reliability of the scale was approved based on the internal consistency coefficient of 0.86. All the items are positively worded and rated using a 5-point Likert scale (1 = absolutely disagree and 5 = strongly agree). The total score of the questionnaire would range from 14 to 70.

In an attempt to examine Chinese high-school students' FLE, Li et al. (2018) developed the Chinese version FLE scale based on the original questionnaire which was proposed by Dewaele and MacIntyre (2014). The Chinese questionnaire included 11 items and the internal consistency of the scale was approved with a Cronbach alpha coefficient of 0.82.

### Individual and Interpersonal Factors Affecting FLCA and FLE

Following the development of FLE scale, Dewaele and MacIntyre (2014) conducted a seminal study to examine the nature of the relationship between FLCA and FLE in 1,740 foreign language learners with different personal characteristics. The research findings demonstrated that there was a significantly positive relationship between FLE and age, FL proficiency, academic degrees, multilingualism, classroom tasks, as well as teachers' support. They also emphasized that such factors had a significantly negative impact on FLCA.

Findings from Dewaele et al. (2018) study demonstrated that learners' age had a significant influence on FLE, but it had no effects on FLCA. They further declared that there is a significant association between language proficiency and FLE and FLCA; the higher the learners' level of FL, the higher FLE scores and the lower FLCA scores were reported. Finally, they asserted that attitudes toward the target language and also toward teachers have significant impacts on FLE, yet moderate effects on FLCA.

It is always crucial for teachers and even material developers to be aware of individual differences (e.g., emotions) among language learners and to design FL courses so that all sorts of learners can benefit from the content in a supportive and low-stress atmosphere (Benesch, 2017). Similarly, Gkonou et al. (2020) argued that there is a strong and direct relationship between teachers' motivation and learners' motivation in the classroom. They claimed that teachers' enthusiasm and empathy can help language learners perceive lower anxiety and experience higher levels of self-confidence and autonomy while performing classroom tasks (Derakhshan et al., 2021b).

## Suggestions for Future Research

Previous studies highlighted the significance of positive and negative emotions in language learning outcomes; it is hence imperative to employ reliable and valid instruments to conduct appropriate and generalizable studies. FLCA scale and FLE scale are among the well-known and highly reliable instruments, respectively. These two questionnaires have been translated into other languages; however, there is still the need to translate them into more languages such as Persian and also to perform psychometric analysis on the modified versions of these instruments.

It is also noteworthy that language learning is a dynamic process, particularly the emotional aspect of FL learning (Li et al., 2018; Dewaele and Dewaele, 2020). Therefore, it is necessary to conduct more longitudinal studies to ensure the reliability and generalizability of the respective findings. For instance, Elahi Shirvan and Taherian (2021) investigated the improvement of FLE and FLCA among 367 undergraduate students. The data were collected using FLE Scale and FLCA Scale from October to November 2016. They concluded that there is a substantial but negative relationship between the growth of FLE and FLCA.

Arnold and Fonseca (2007) argued that the learning environment (including teachers and peers' support) should direct learners toward facing and resolving educational challenges, and eventually FL attainment. In a similar vein, Nemati et al. (2020) proposed that interesting classroom tasks, sociocultural and intrinsic factors, as well as positive attitudes to teachers and fellow classmates, have led to a significant improvement of FLE and moderate reduction of FLCA (in particular, speaking-induced anxiety) among Iranian learners with intermediate and upper-intermediate English proficiency levels. However, Dewaele et al. (2018) concluded that learner-related variables can have more significant impacts on language learning compared to external factors (language classroom, peers, and teachers). Consequently, it is recommended to conduct more in-depth studies on the effectiveness and significance of teachers' and peers' role in the improvement of FLE and reduction of FLCA.

## Author Contributions

JS read contemporary scholarly articles and books and presented the effectiveness of foreign language enjoyment and foreign language classroom anxiety on learners' engagement and attainment.

## Conflict of Interest

The author declares that the research was conducted in the absence of any commercial or financial relationships that could be construed as a potential conflict of interest.

## Publisher's Note

All claims expressed in this article are solely those of the authors and do not necessarily represent those of their affiliated organizations, or those of the publisher, the editors and the reviewers. Any product that may be evaluated in this article, or claim that may be made by its manufacturer, is not guaranteed or endorsed by the publisher.
